# Multimorbidity Patterns and Periodontal Diseases in a French Hospital-Based Dental School: A Retrospective Cross-Sectional Study

**DOI:** 10.3390/jcm13226631

**Published:** 2024-11-05

**Authors:** Blandine Robert, Maxime Bonjour, Brigitte Grosgogeat, Kerstin Gritsch

**Affiliations:** 1 Faculté d’Odontologie, Université Claude Bernard Lyon 1, 69008 Lyon, France; blandine.robert@univ-lyon1.fr (B.R.); brigitte.grosgogeat@univ-lyon1.fr (B.G.); 2 Department of Periodontology, Service d’Odontologie, Hospices Civils de Lyon, 69007 Lyon, France; 3 Service de Biostatistique-Bioinformatique, Pôle Santé Publique, Hospices Civils de Lyon, 69003 Lyon, France; 4 Équipe Biostatistique-Santé, Laboratoire de Biométrie et Biologie Évolutive, UMR CNRS 5558, 69100 Villeurbanne, France; 5 Faculté de Médecine Lyon Est, Université Claude Bernard Lyon 1, 69008 Lyon, France; 6 Laboratoire des Multimatériaux et Interfaces, UMR CNRS 5615, 69622 Villeurbanne, France; 7 Department of Clinical Research, Service d’Odontologie, Hospices Civils de Lyon, 69007 Lyon, France

**Keywords:** prevalence, pathology, periodontal diseases, dental school, multimorbidity

## Abstract

**Objectives:** To assess the prevalence of systemic pathologies associated with periodontal diseases to determine multimorbidity patterns and risk factors for periodontal care. **Methods:** A single-center cross-sectional study analyzed patient records from 1 January 2019 to 31 December 2021 at the Department of Periodontology, part of the Dental Service of a hospital-based dental school in Lyon, France. The anonymized data included patient-reported medical history (questionnaire) and billing for periodontal procedures. Data were compared between patients in the Department of Periodontology and from Other Departments of the Dental Service. The association between clinically relevant pathologies, as defined as multimorbidity, and admission in the Department of Periodontology were assessed with logistic regression. Prevalences between the Department of Periodontology and the Other Departments of the Dental Service were compared with chi-squared tests. Relationship among pathologies was described through correlation analysis. Statistical analyses were conducted using R software with a significance level set at *p* < 0.05. **Results:** This study included records of 20,945 patients in the Dental Service with 1205 periodontal procedures performed in the Department of Periodontology. Patients admitted for periodontal care were older and mostly female (*p*-value < 0.001). The most frequent systemic pathologies were hypertension and diabetes in the Department of Periodontology. Hypertension associated with diabetes was the most frequent multimorbidity pattern, while the most frequent triad of multimorbidity was hypertension/diabetes/cardiac rhythm disorders. Patients with diabetes had a 1.49 times higher likelihood of admission to the Department of Periodontology (OR = 1.49 [1.20; 1.86]), with age also being a significant risk factor but with a smaller effect size (OR = 1.02 [1.01; 1.03]). **Conclusions:** Within the limitations of this study, hypertension, diabetes, cardiac rhythm disorders, and chronic renal insufficiency were identified as prevalent multimorbidity in the Department of Periodontology. Multimorbidity including diabetes seems to constitute a risk factor requiring periodontal care.

## 1. Introduction

Periodontal diseases are pathological conditions affecting the periodontium, which refers to the supporting structures around the tooth. This includes the gingival tissues, alveolar bone, cementum, and periodontal ligament. Gingivitis, a reversible form of periodontal disease affecting up to 90% of the population, can progress to periodontitis, a chronic and irreversible inflammatory condition. This progression is primarily associated with a dysbiosis of the subgingival biofilm, where pathogenic bacteria outcompete commensal microbial communities and whose penetration into tissues triggers the host’s immune response. Dysregulation of the host immune and inflammatory response ultimately leads to destruction of the periodontium and potential tooth loss [1]. The detrimental consequences of periodontal diseases were reported on oral health and quality of life [2], as well as the association of periodontal diseases with various systemic pathologies [3,4,5,6], and the improvements in systemic health related to periodontal treatments [7]. There are many data regarding the bidirectional association between periodontal diseases and specific systemic chronic pathologies, such as cardiovascular diseases, diabetes, chronic inflammatory bowel diseases, renal insufficiency, Alzheimer’s disease, and rheumatoid arthritis [8,9,10,11,12,13]. Moreover, the impact of the periodontal treatment on inflammation and the course of systemic pathologies has been highlighted, especially in diabetes [9,14]. Two mechanisms have been investigated to explain this association: systemic inflammation and bacteremia [8,15,16]. Despite the significant public health impact of periodontal diseases and their association with systemic pathologies, data regarding the impact of these diseases on patients with multimorbidity (defined as the coexistence of two or more chronic systemic pathologies) remain limited [17,18]. This critical research gap emphasizes the need to understand multimorbidity in relation to periodontal disease, as it is insufficiently studied and may complicate the care pathway. Patients with multimorbidity may require tailored care strategies that consider the interactions between their different systemic pathologies and oral health (rather than treating each condition in isolation), ensuring personalized treatment plans. Moreover, the differences in public health policies between countries may lead to variations in healthcare pathways and access for periodontal care among patients with multimorbidity. This underscores the importance of studying these factors at different scales. For instance, a recent study conducted in a dental school in the Netherlands revealed significant findings in this area, which justifies the investigation in a hospital-based dental school in France, considering the different public health context [17].

Therefore, it is crucial to determine multimorbidity patterns among patients undergoing treatment for periodontal disease in order to identify the specific coexistence of pathologies that may represent additional risk factors for periodontal disease. This approach could help determine whether multimorbid patients experience a cumulative or potentiated risk of periodontal disease beyond that associated with each individual condition. In this context, the aims herein were to assess the prevalence of systemic pathologies associated with periodontal diseases and to determine multimorbidity patterns that constitute risk factors for periodontal care.

## 2. Materials and Methods

### 2.1. Study Design, Population, and Data Collection

A single-center retrospective cross-sectional study was conducted in the Department of Periodontology, part of the Dental Service of a hospital-based dental school of the Hospices Civils de Lyon (HCL, Lyon, France). Two categories of records were included: those of adult patients (≥18 years) who attended their first consultation at the Dental Service between 1 January 2019 and 31 December 2021 and completed a medical questionnaire, and among these, records of patients admitted to the Department of Periodontology who were billed with a periodontal care code between 1 January 2019 and 31 December 2021. This study was approved by the Institutional Review Board Comité d’éthique of the HCL (approval number 24-5320); the data were considered anonymized, and no informed consent was required. This study was conducted in accordance with the STROBE guidelines [19].

The Dental Service is organized into various departments, each specializing in different dental fields such as Periodontology, Prosthetics, and Conservative Dentistry for example. When a periodontal disease (such as gingivitis, periodontitis, or, less commonly, peri-implantitis due to presence in the Department of Implantology) is identified during a patient’s care within the Dental Service, the patient is referred to the Department of Periodontology.

During the initial consultation and the potential subsequent consultations, each patient was required to complete a medical questionnaire during an interview with a dental surgeon. The patients’ medical histories were self-reported, and responses were verified through discussion and by corroborating the responses with clinical records, thereby minimizing potential biases associated with self-reporting. The completed medical questionnaire was then added to the patient’s electronic health record. Medical questionnaires performed during the study period were extracted, and duplicates were removed (the most recent questionnaire was defined as the reference since it was considered the most recently updated). Information on sex (male/female), age, current smoking status (yes/no), and patients’ self-reported medical history was collected. To mitigate the risk of missing data, all questions about systemic pathologies were structured with binary (yes/no) options and were reviewed by a dental surgeon. The other parameters (sex, age, and current smoking status) were completed during the initial consultation. Eight categories of pathologies, based on their significant associations with periodontal disease, were extracted: hypertension, cardiac rhythm disorders, heart failure (these three constituting cardiovascular diseases), diabetes (insulin-dependent or non-insulin-dependent), Crohn’s disease, chronic renal insufficiency, Alzheimer’s disease, and rheumatoid arthritis. A patient with multimorbidity was defined as having at least two of these conditions. Moreover, the extracted billed periodontal procedures of the Department of Periodontology were (i) consultation, (ii) periodontal charting, (iii) scaling and root planning, and (iv) surgical periodontal therapy. The collected characteristics were anonymized and compared between patients admitted to the Department of Periodontology and those admitted to the Other Departments of the Dental Service.

### 2.2. Statistical Analyses

Variables were expressed as count and percentage, and as mean and standard deviation (SD). Comparisons of prevalence were performed using Pearson’s chi-squared test with continuity correction. The analysis of coexisting pathologies was carried out by modeling a set visualization diagram for the Department of Periodontology and the Other Departments of the Dental Service. Multivariate logistic regression analysis was performed to assess the effect of the association between the clinically relevant variables (hypertension, cardiac rhythm disorders, heart failure, diabetes, Crohn’s disease, chronic renal insufficiency, Alzheimer’s disease, rheumatoid arthritis, tobacco, age). All the available data were included to study multimorbidity patterns. The effect of multimorbidity on the reduction in the occurrence of presence in the Department of Periodontology was assessed by a likelihood ratio test and quantified through the adjusted odds ratio (OR) with the associated 95% confidence interval [95% CI]. This test evaluates the significance of individual predictors in the model, providing clearer insights into their contributions to the outcomes being studied. Furthermore, a multimorbidity pattern was modeled after the calculation of the Pearson correlation coefficient (r) and 95% CI to assess relationships between the pathologies studied pairwise in the Department of Periodontology and in the Other Departments of the Dental Service. All analyses were performed using R software version 4.2.0 (R foundation for statistical computing, Vienna, Austria), and the significance level was set at *p*-value < 0.05 [20].

## 3. Results

### 3.1. Characteristics of the Study Population

**Figure 1:**
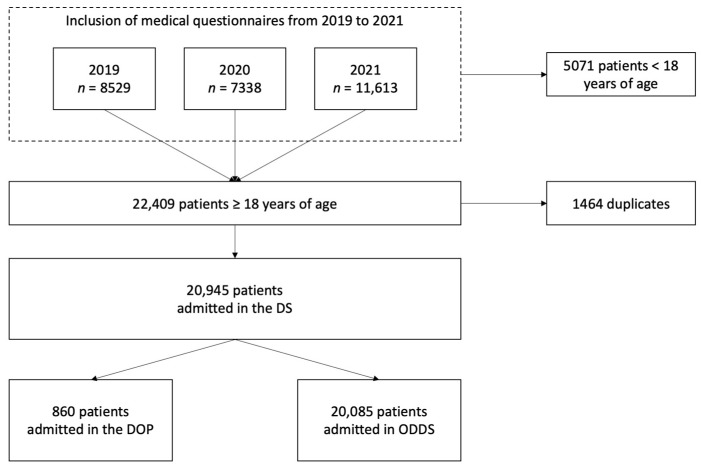
Flowchart of the steps to reach the final study population in the Department of Periodontology and in the Other Departments of the Dental Service. DS: Dental Service, DOP: Department of Periodontology, ODDS: Other Departments of the Dental Service.

This study included the records of 20,945 patients admitted to the Dental Service between 1 January 2019 and 31 December 2021 (Figure 1). Among them, 860 patients were admitted to the Department of Periodontology within the same period. For all the variables considered, there were no missing data. The mean (SD) age of the patients in the Department of Periodontology was 52.9 (14.2) years old, whereas it was 45.6 (17.9) years old in the Other Departments of the Dental Service. Patients in the Department of Periodontology were significantly older than patients in the Other Departments of the Dental Service (*p* < 0.001). There was also a significantly higher proportion of females in the Department of Periodontology (55.8%) than in the Other Departments of the Dental Service (48.3%, *p* < 0.001). No significant difference was found regarding the proportion of current smokers between patients in the Department of Periodontology (26.3%) and Other Departments of the Dental Service (27.9%; Table 1).

### 3.2. Prevalence of Systemic Pathologies

**Figure 2:**
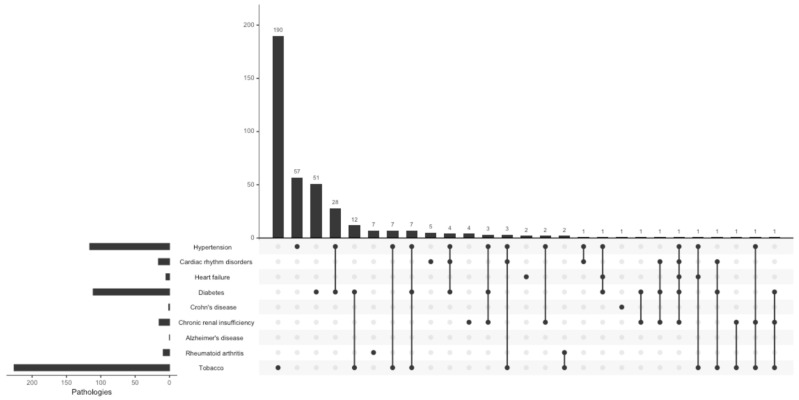
Set visualization diagram of coexistence of pathologies in the Department of Periodontology.

**Figure 3:**
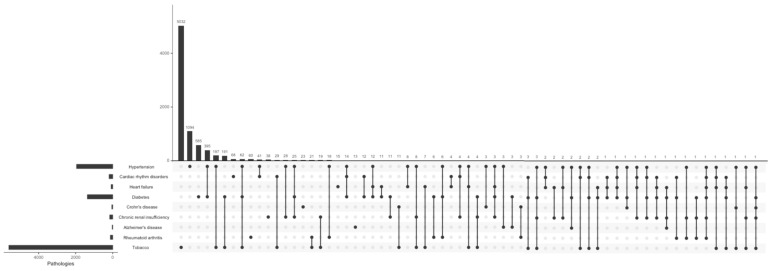
Set visualization diagram of coexistence of pathologies in the Other Departments of the Dental Service.

The most frequently reported systemic pathologies in the Department of Periodontology were hypertension (13.4%) and diabetes (12.9%), as well as in the Other Departments of the Dental Service (9.7% and 6.8%, respectively; Table 1 and Figure 2 and Figure 3). There was a higher proportion of hypertension (*p* < 0.001), cardiac rhythm disorders (*p* = 0.014), diabetes (*p* < 0.001), and chronic renal insufficiency (*p* = 0.004) in patients admitted to the Department of Periodontology compared to patients in the Other Departments of the Dental Service. On the other hand, there was no significant difference between the proportions of heart failure, Crohn’s disease, Alzheimer’s disease, and rheumatoid arthritis observed in the Department of Periodontology and in the Other Departments of the Dental Service. A significant difference was found regarding the proportion of currently smoking patients with diabetes admitted to the Department of Periodontology compared to the Other Departments of the Dental Service (*p* = 0.011); there was no significant difference found in the proportions regarding the smoking status for all the other studied pathologies (Table 1).

### 3.3. Multimorbidity

**Figure 4:**
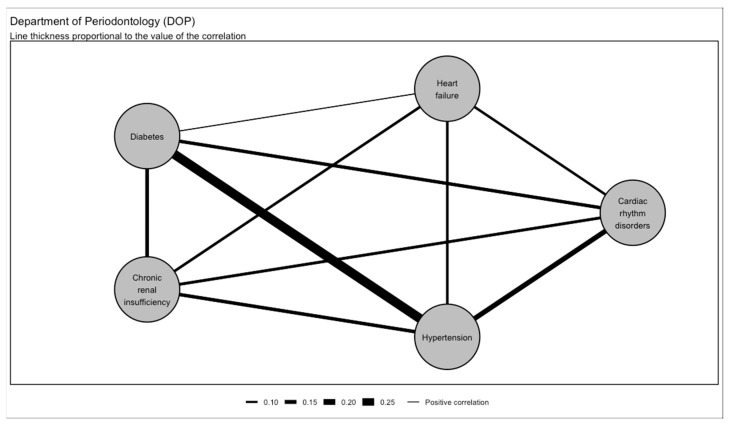
Multimorbidity network in the Department of Periodontology (DOP).

**Figure 5:**
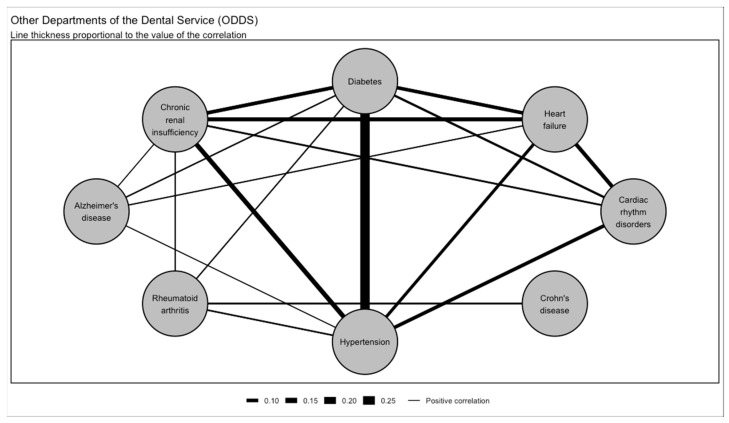
Multimorbidity network in the Other Departments of the Dental Service (ODDS).

Multimorbidity was examined in the Department of Periodontology (Figure 2 and Figure 4) and in the Other Departments of the Dental Service (Figure 3 and Figure 5). Hypertension associated with diabetes was the most common multimorbidity found in the Department of Periodontology as well as in the Other Departments of the Dental Service. The proportion of hypertension associated with diabetes was significantly greater in the Department of Periodontology compared to the Other Departments of the Dental Service in both non-current smokers (3.3% and 2.0%, respectively, *p* = 0.012) and current smokers (0.8% and 0.3%, respectively, *p* = 0.026; Figure 2 and Figure 3).

Among non-current smokers, hypertension associated with chronic renal insufficiency was the second most frequent dyad pattern of multimorbidity in the Department of Periodontology (0.2%), whereas in the Other Departments of the Dental Service, it was hypertension associated with cardiac rhythm disorders (0.1%). Among current smokers, hypertension associated with chronic renal insufficiency was the second most frequent multimorbidity in the Other Departments of the Dental Service (0.04%). The most frequent triad pattern of multimorbidity among non-current smokers in the Department of Periodontology was hypertension associated with diabetes and cardiac rhythm disorders (0.5%), followed by hypertension associated with diabetes and chronic renal insufficiency (0.4%). Conversely, in the Other Departments of the Dental Service, the most frequent combination was hypertension associated with diabetes and chronic renal insufficiency (0.1%), followed by hypertension associated with diabetes and cardiac rhythm disorders (0.07%). The Department of Periodontology did not allow any patients who were current smokers and had at least three pathologies (e.g., no currently smoking patients with ≥3 pathologies presented at the Department of Periodontology). Moreover, the number of multimorbidity combinations was lower in the Department of Periodontology population compared to the Other Departments of the Dental Service population (Figure 2 and Figure 3).

When compared to the Other Departments of the Dental Service, in the Department of Periodontology, there was a stronger correlation between diabetes and hypertension (r = 0.29, 95% CI [0.28; 0.30]), hypertension and cardiac rhythm disorders (r = 0.17, 95% CI [0.16; 0.18]), diabetes and chronic renal insufficiency (r = 0.13, 95% CI [0.12; 0.14]), diabetes and cardiac rhythm disorders (r = 0.12, 95% CI [0.11; 0.13]), chronic renal insufficiency and cardiac rhythm disorders (r = 0.11, 95% CI [0.10; 0.12]), and hypertension and heart failure (r = 0.10; Figure 4 and Figure 5).

In the univariate logistic regression analysis, it was found that the risk of being admitted to the Department of Periodontology was greater in patients with hypertension (OR = 1.45, 95% CI [1.19; 1.78]), cardiac rhythm disorders (OR = 1.97, 95% CI [1.18; 3.30]), diabetes (OR = 2.03, 95% CI [1.65; 2.50]), or chronic renal insufficiency (OR = 2.25, 95% CI [1.32; 3.84]). However, the logistic regression adjusted on hypertension, cardiac rhythm disorders, heart failure, diabetes, Crohn’s disease, chronic renal insufficiency, Alzheimer’s disease, rheumatoid arthritis, smoking status, and age showed that only age (OR = 1.02, 95% CI [1.01; 1.03]) and diabetes (OR = 1.49, 95% CI [1.20; 1.86]) were associated with a greater probability of being admitted to the Department of Periodontology. Smoking status was not a risk factor for being admitted to the Department of Periodontology in the studied population (Table 2).

## 4. Discussion

The present study found that the most prevalent systemic pathologies reported in the Department of Periodontology and in the Other Departments of the Dental Service were hypertension, cardiac rhythm disorders, diabetes, and chronic renal insufficiency. Proportions of these pathologies were significantly different between the Department of Periodontology and the Other Departments of the Dental Service. The most common multimorbidity pattern reported herein was the dyad pattern composed of hypertension and diabetes; this dyad was also reported with additional systemic pathologies comprising cardiac rhythm disorders or chronic renal insufficiency. However, only age and diabetes were found to be associated with being admitted to the Department of Periodontology.

The important proportion of patients with hypertension reported herein aligns with the reported association between periodontal diseases and hypertension [8,21,22,23]. Similarly, in the present study, a greater proportion of diabetes was found in the Department of Periodontology compared to the French overall population [24]. These results align with the bidirectional relationship between periodontal diseases and diabetes described in the literature [9].

The present study found no significant difference between the proportion of heart failure, Crohn’s disease, Alzheimer’s disease, and rheumatoid arthritis in the Department of Periodontology and in the Other Departments of the Dental Service. However, the low prevalence of these pathologies in the overall population should be considered [25,26,27,28]. For instance, the prevalence of inflammatory bowel diseases (IBDs) is estimated at 0.42% in the French overall population, but the prevalence of Crohn’s disease alone is unknown [26]. Nevertheless, it is surprising to note the lack of the elevated prevalence of these pathologies in the Department of Periodontology since several studies reported bidirectional relationships between these systemic pathologies and periodontitis [8,10,12,13].

Additionally, dental surgeons seem to have integrated periodontitis as the sixth diabetes complication, and potential selection biases among caregivers should be considered as they may provide greater caution in directing patients with diabetes to periodontal care [29]. In the French healthcare system, Public Insurance covers the initial periodontal therapy for patients with diabetes registered as a “long-term health condition”. This complete coverage may likely contribute to the greater number of patients with diabetes found in the Department of Periodontology. Furthermore, the extension of this coverage to additional long-term health conditions, including some cardiovascular diseases as well as rheumatoid arthritis, could improve awareness, but above all, acceptance and adherence to periodontal care. Moreover, further recognition that, in multimorbid populations, the impact of periodontitis can be as substantial as comorbid diabetes mellitus will likely drive better management strategies and help curb rising medical expenditures [30].

The findings of the present study are in accordance with those of a recent study that assessed the correlation between periodontal diseases and the presence of multimorbidity [17]. Beukers et al. indicated that patients with periodontal diseases are more likely to present with other systemic pathologies. However, unlike herein, their study reports higher adjusted odds for presenting with up to four systemic pathologies in patients with periodontal diseases. Although some stronger correlations between pairwise multimorbidity patterns were observed in the Department of Periodontology, only the presence of diabetes among multimorbidity had a statistically significant influence on being admitted to the Department of Periodontology.

The dyad pattern of multimorbidity composed of hypertension and diabetes was the most frequent pattern in both the Department of Periodontology and the Other Departments of the Dental Service, with a significantly greater proportion in the Department of Periodontology (among non-smokers and smokers). The frequent coexistence of these two systemic pathologies aligns with previous findings that reported diabetes and hypertension as the most frequently encountered pathologies in patients admitted to a dental hospital in Saudi Arabia [31]. Furthermore, the most frequent multimorbidity pattern in the Department of Periodontology was the triad composed of hypertension, diabetes, and cardiac rhythm disorders; it has been reported that periodontal diseases are a risk factor for diabetes and cardiovascular diseases [32]. Dental surgeons should be cautious of the frequent coexistence of these pathologies and their interconnections [33]. Moreover, interdisciplinary collaboration is essential for comprehensive patient care; screening and regular follow-up protocols should be implemented for high-risk patients. Raising awareness among dental surgeons and specialists regarding the associations between periodontal diseases and systemic pathologies will lead to more effective treatment strategies. Additionally, improving access to periodontal care and emphasizing the importance of good oral hygiene and preventive treatments will enhance patient care and promote holistic approaches to oral and systemic health.

In the present study, a significantly higher proportion of women in the Department of Periodontology was observed than in the Other Departments of the Dental Service contrary to the results of Beukers et al. [17]. This reinforces the hypothesis of an influence of sex steroid hormones on periodontal tissues [34]. On the other hand, studies have shown that women use more healthcare services than men, suggesting a greater inclination to adhere to periodontal care, for example [35].

This observational study has limitations in part due to the biases inherent to its single-center and retrospective design. Moreover, reliance on patient self-reported health may introduce biases and inaccuracies. However, confirming patient-declared pathologies through interviews with dental professionals allowed us to partially mitigate this risk. While it is conceivable that some patients in the Other Departments of the Dental Service group may have had undiagnosed periodontal disease, this hypothesis is quite limited given the structured nature of the care pathway in the Dental Service. In addition, it was observed that smokers do not face an elevated risk of admission in the Department of Periodontology compared to non-smokers. However, tobacco is acknowledged as a risk factor for periodontitis [36]. This discrepancy may stem from factors such as the quantity of tobacco consumed or the potential inclusion of former smokers, which were not accounted for in this retrospective study. Moreover, the used codes lack the capacity to assess the severity of periodontitis or the studied systemic pathologies. This study lacks statistical power, in part due to the limited number of patients with multimorbidity, which might limit the identification of subtle associations between periodontal diseases and multimorbidity. In addition, a confounding bias may persist since no adjustment was initially performed for factors such as addictive behaviors, socioeconomic status, dietary habits, and drug treatments. Furthermore, the study period spanned the different phases of the COVID-19 pandemic, from its initial spread (without vaccines) to the post-vaccine-authorization period. One of the limitations of this study could lie in the significant changes in the number of consultations, especially for patients with systemic pathologies, considered to be at-risk for COVID-19. However, Pearson’s chi-square analysis with continuity correction was performed to assess this potential limitation, and no significant impact was found between each Period (Period 0 from 1 January 2019 to 15 August; Period 1 from 16 August 2019 to 16 March 2020; Period 2 from 16 June 2020 to 31 December 2020; Period 3 from 1 January 2021 to 30 June 2021; and Period 4 from 1 July 2021 to 31 December 2021). Finally, the present results should be interpreted with caution since the Department of Periodontology is located in Lyon, one of the five largest cities in France, where access to healthcare is easier than in rural areas [37]. This highlights the importance of considering the specific population studied when interpreting prevalence data and that further studies are needed to explore the complex relationship between periodontal diseases and multimorbidity.

Future multicentric studies should be conducted to improve the generalizability of findings related to multimorbidity and periodontal diseases. In addition, employing longitudinal designs will allow researchers to monitor changes over time, thereby clarifying the temporal relationships between these conditions and potentially identifying causal links. It is also important to expand the analysis to include confounding factors such as lifestyle behaviors, socioeconomic status, and medication use. Moreover, evaluating the severity of both periodontal and systemic conditions could offer valuable insights into their interactions. Finally, conducting more detailed investigations into smoking status, particularly regarding their duration and intensity, as well as vaping, is essential for understanding their effects on periodontal health and their interactions with other risk factors.

## 5. Conclusions

This study highlights the prevalence of hypertension, diabetes, cardiac rhythm disorders, and chronic renal insufficiency among patients in the Department of Periodontology, which allowed us to identify the most prevalent multimorbidity patterns. Multimorbidity, including diabetes, seems to constitute a risk factor for periodontal care and thus requires more targeted referrals, considering the medical questionnaire. However, these findings must be interpreted with caution due to significant limitations inherent in the study design. Further studies are needed as identifying the cumulative risk factors associated with periodontal disease in multimorbid patients is crucial. This study underscores the necessity for improved patient management and the implementation of integrated approaches to oral and systemic health. These findings can guide healthcare policies and the development of comprehensive care strategies that enhance patient outcomes and healthcare efficiency.

## Figures and Tables

**Table 1:** Characteristics of the patients and prevalence of systemic pathologies in the Department of Periodontology (DOP) and in the Other Departments of the Dental Service (ODDS).

Characteristics	DOP N = 860	ODDS N = 20,085	*p*-Value
Age	52.9 (14.24)	45.6 (17.87)	<0.001
Sex			
Female	480 (55.8%)	9713 (48.3%)	<0.001
Male	380 (44.2%)	10,372 (51.7%)	
Current smokers	226 (26.3%)	5605 (27.9%)	0.316
Hypertension			
All	116 (13.4%)	1948 (9.7%)	<0.001
Current smokers	19 (2.2%)	282 (1.4%)	0.072
Cardiac rhythm disorders			
All	16 (1.8%)	191 (0.95%)	0.014
Current smokers	4 (0.5%)	38 (0.2%)	0.167
Heart failure			
All	5 (0.6%)	81 (0.40%)	0.598
Current smokers	1 (0.1%)	16 (0.08%)	1
Diabetes			
All	111 (12.9%)	1365 (6.8%)	<0.001
Current smokers	21 (2.4%)	271 (1.3%)	0.011
Crohn’s disease			
All	1 (0.1%)	44 (0.22%)	0.794
Current smokers	0 (0%)	13 (0.06%)	0.962
Chronic renal insufficiency			
All	15 (1.7%)	157 (0.78%)	0.004
Current smokers	3 (0.3%)	36 (0.2%)	0.468
Alzheimer’s disease			
All	0 (0%)	23 (0.11%)	0.640
Current smokers	0 (0%)	1 (4.9 × 10^−5^ %)	1
Rheumatoid arthritis			
All	9 (1.0%)	121 (0.60%)	0.161
Current smokers	2 (0.2%)	23 (0.1%)	0.633

Note: Values represent count (%) or mean in years (±SD).

**Table 2:** Logistic regression analysis of multimorbidity within the Department of Periodontology (DOP).

	OR Univariate [95% CI]	OR Adjusted † [95% CI]	*p*-Value Adjusted
Hypertension	1.45 [1.19; 1.78]	0.84 [0.68; 1.05]	0.132
Cardiac rhythm disorders	1.97 [1.18; 3.30]	1.30 [0.77; 2.20]	0.325
Heart failure	1.44 [0.58; 3.57]	0.78 [0.31; 1.96]	0.594
Diabetes	2.03 [1.65; 2.50]	1.49 [1.20; 1.86]	<0.001
Crohn’s disease	0.53 [0.07; 3.85]	0.53 [0.07; 3.88]	0.533
Chronic renal insufficiency	2.25 [1.32; 3.84]	1.49 [0.86; 2.58]	0.153
Alzheimer’s disease	NA	NA	NA
Rheumatoid arthritis	1.74 [0.88; 3.45]	1.29 [0.65; 2.57]	0.462
Tobacco	0.92 [0.79; 1.08]	1.08 [0.92; 1.27]	0.306
Age	1.02 [1.01; 1.03]	1.02 [1.01; 1.03]	<0.001

† Logistic regression adjusted on hypertension, cardiac rhythm disorders, heart failure, diabetes, Crohn’s disease, chronic renal insufficiency, Alzheimer’s disease, rheumatoid arthritis, tobacco, and age. NA: not applicable, OR: odds ratio, CI: confidence interval.

## Data Availability

The data presented in this study are available on request from the corresponding author due to the data sharing policy of the institution.
